# Adequate use of asthma inhalation medication in children: more involvement of the parents seems useful

**DOI:** 10.1186/1756-0500-2-129

**Published:** 2009-07-13

**Authors:** Johannes HJM Uijen, Yannick JW van Uijthoven, Johannes C van der Wouden, Patrick JE Bindels

**Affiliations:** 1 Department of General Practice, Room Ff305, Erasmus MC – University Medical Center Rotterdam, PO Box 2040, 3000 CA Rotterdam, The Netherlands

## Abstract

**Background:**

Asthma and other chronic airway diseases can be effectively treated by inhaler therapy. Inhaler therapy depends on appropriate use of the inhaler. This study evaluates the knowledge among Dutch children and their parents regarding asthma inhaler therapy and appropriateness of its use.

**Findings:**

Five general practices selected all children aged 0 to 12 years on asthma inhalation medication. Children demonstrated inhaler use and were interviewed with their parents.

46 subjects were enrolled; mean age 5.5 years (SD 3.4) years; 26 (57%) were boys. Of the children using one inhaler only, 70% used the inhaler as indicated and of those using more than one inhaler 46%. On average 2.6 mistakes were made during demonstration of the technique, and 2 mistakes were reported in the interview. In total, 87% of the parents decided when and how the inhaler had to be used. Spacer cleaning was performed correctly by 49%; 26% reported a correct way of assessing how many doses were remaining.

**Conclusion:**

Dutch children make essential mistakes related to inhaler use that are easy to avoid. We recommend a better explanation and demonstration of the technique, and recommend involvement of the parents during instruction.

## Introduction

Asthma and other chronic airway diseases can be effectively treated by inhaler therapy [1]. Inhaler devices come in a variety of types, such as metered dose inhalers (MDI) or dry powder inhalers (DPI). Irrespective of the type of inhaler device used, the outcome of inhaler therapy largely depends on appropriate use of the inhaler.

Appropriate use primarily involves the correct inhalation technique. A poor inhalation technique reduces drug deposition in the lungs [2]; moreover, the more mistakes made in the inhalation technique the lower the beneficial effect on lung function [3]. From adults it is known that 89% of the patients make at least one mistake in the inhalation technique [4]. Also children face difficulties in using an inhaler. A study among Taiwanese children showed that none of the children had a perfect inhalation technique [5], a Dutch study reported that even after instructing children the overall inhalation technique remained poor [6]. In contrast, a study from Malta showed that only 17% of children using an MDI with a spacer device had a poor technique [7].

Appropriate inhaler use also involves actual use compared with the advised regimen of the prescriber. Several studies have shown that, even with adequate inhaler use (between 50 and 80% of prescribed doses), compliance with inhalation corticosteroids (ICS) is far from perfect [8-12]. Overuse of bronchodilators has also been reported and some parents confuse the corticosteroid inhaler (for maintenance therapy) with the bronchodilator inhaler (to be used in case of symptoms) [13,14].

Little is known about the current situation regarding the appropriate use of inhalers by children in the Netherlands. Therefore the purpose of this study is to determine the level of knowledge of children and their parents associated with the correct use of the inhaler. We also wanted to identify inconsistencies between use of inhalers compared with prescriber advice on inhaler use.

## Methods

### Subjects

We included all children aged from 0 to 12 years who had been prescribed inhalation medication in the last three months. For this reason, the electronic patient files were searched for patients who had been prescribed relevant medication using the ATC codes R03A (adrenergics) and R03B (other drugs for obstructive airway diseases, including ICS) [15]. A convenience sample of five Dutch general practices in both rural and urban areas was invited to participate.

### Data collection

The general practitioner (GP) sent parents of the children a letter with an informed consent form, a request to participate, and an answer form that had to be returned to the investigators. Subjects that responded positively were visited at home during the period April to July 2007 by a well-trained investigator who observed the inhalation technique and held a face-to-face interview.

Questions on the inhalation technique were posed to the children themselves if they were aged five years and older; if they were younger the parents answered these questions. Additional general questions were always answered by the parents.

### Assessing appropriate use

The investigator assessed the child's inhalation technique using an inhaler specific checklist adapted from the checklists of the Dutch Asthma Foundation [16,17]. Children were asked to demonstrate their inhalation technique and any mistakes were written down. Essential mistakes were identified [17]. These involved preparing or loading the device prior to inhalation; slow continuous inhalation (MDI) or deep forceful inhalation (DPI); waiting too long before inhaling a spacer after activating the MDI; and incorrect spacer mask use.

In order to exactly compare inhalation technique itself with knowledge on inhalation technique, the investigator administered a second questionnaire after the child had demonstrated the inhalation technique.

Finally the parents were asked how they assessed the number of remaining doses and how they cleaned the device. Both questionnaires can be found in the additional file [see Additional file 1].

### Assessing actual use compared with prescribed daily dose

After the interview, the pharmacy prescription labels of the inhalation medication were collected. With permission of the parents, the information written on the prescription label was copied. If the label was no longer available the parents were asked to provide written consent to obtain the prescription details from the GP.

### Data analysis

All data were analysed with SPSS version 11.0. All analyses were descriptive.

## Results

### Response and inhaler use

All five practices agreed to participate and a total of 162 children were selected from the electronic medical files. A reply was received from 56 subjects (34%), of which 10 (16%) refused to participate. The most frequently mentioned reason for refusal (among responders) was that the inhalation medication was no longer used. The most frequent reason for refusal (among responders) was that the inhalation medication was no longer used. Two subjects refused because of a stressful situation at home. The 46 subjects (28%) enrolled had a mean age of 5.5 (SD 3.4) years. Thirty (65%) were aged five years or older. Twenty-six (57%) of the subjects were boys. Twelve (26%) of the children lived in an urban region, while 34 (74%) lived in a rural area. Most children (n = 41; 89%) used an MDI in combination with a spacer device, four children (9%) used a DPI and only one child (2%) used an MDI without a spacer. Thirty-three children (72%) used a bronchodilator in combination with an ICS, the remaining children used either a bronchodilator (n = 12; 26%) or an ICS (n = 1; 2%).

### Inhalation technique

Because two very young children refused to demonstrate their inhalation technique, demonstration data are available for only 44 of the 46 children. Table 1 summarizes the three most frequently made mistakes during the demonstration of the technique and the three most frequent incorrect answers. One child made no mistakes during the demonstration.

**Table 1:** Essential mistakes during demonstration of inhalation technique and in questionnaire (n = 44)

*Type of mistake made in demonstration of technique*	*n*	*(%)*
Forgot to shake inhaler before use	9	(21)
Waiting >5 sec before inhaling spacer after MDI activation	8	(18)
Not pressing spacer mask on face	8	(18)

*Type of mistake made in questionnaire on technique*		

Activating MDI twice before inhaling through spacer, when two doses are needed	19	(43)
Not rinsing the mouth after using ICS inhaler	4	(9)
Making less than five inhalations through spacer	3	(7)

Not shaking the inhaler before use was the most frequently made mistake (n = 9; 20%) during the demonstration; and "When I need two doses, I can activate MDI twice before starting to inhale through spacer" was the most frequently noted incorrect answer (n = 19; 43%). Each child made on average 2.6 mistakes (range: 0 – 7) in demonstrating, and on average 2 mistakes (range: 0 – 5) were noted on the questionnaire.

### Actual use of inhalers compared with prescribed use

Pharmacy prescription labels were available from 32 children. Twenty of the 32 children (63%) used the inhaler as indicated on the prescription label. All children having one inhaler used their inhaler as indicated on the prescription label. Of the children having two inhalers, only 39% used both their inhalers as indicated on the label. The following mistakes were made: three children used their bronchodilator 'as needed' instead of daily. Five children used their bronchodilator daily instead of 'as needed'. Four children were using their ICS 'as needed' and one child did not use the prescribed bronchodilator.

### Education

Of all parents, 42 (91%) had received some instruction about the inhalation technique; this instruction was clear for 41 of them. Of these 42 parents, 19 (45%) had received instruction at the pharmacy, 11 (26%) at the general practice, 7 (17%) at the hospital, 4 (10%) had more than one source of instruction, and 1 (2%) had received instruction from friends or family. There was no relationship between the different sources of education and mistakes related to inhalation technique or therapy adherence.

### Decision concerning inhaler use

In total, 87% of the parents decided when and how the inhaler had to be used. The mean age of their total of 40 children was 4.9 (SD 3.2) years. The six children, who decided themselves when and how they used the inhaler, had a mean age of 8.5 (SD 1.8) years. There was no significant difference between both groups regarding mistakes.

### Inhaler management

Table 2 summarizes the parental reports on assessing an inhaler for remaining doses of the drug. We considered the following categories to be correct: looking on counter of inhaler; spraying on dark background; if inhaler floats in water, it is empty; and counting remaining doses with agenda. The reports of 12 of the 46 subjects (26%) were correct.

**Table 2:** Parental report on assessing remaining doses (n = 46)

Method used	*n*	*(%)*
*Correct*		
Looking on counter	3	(7)
Spraying against dark background	4	(9)
If inhaler floats in water, it is empty	2	(4)
Counting remaining doses using agenda	3	(7)
*Incorrect*		
Feeling inhaler weight while shaking	9	(20)
Listening to inhaler while shaking	4	(9)
Spraying in the air	13	(28)
If inhaler sinks in water, it is empty	1	(2)
Not assessing at all	7	(14)

*Total*	46	(100)

Table 3 shows the parental reports on cleaning the spacer. We considered only cleaning the spacer with soap, and letting it dry in the air to be correct. Of the 41 subjects (49%) using a spacer, 20 (49%) reported to clean it correctly.

**Table 3:** Parental report on cleaning spacer (n = 41)

Method used	*N*	*(%)*
*Correct*		
Soaping inhaler, dry in air	20	(49)
*Incorrect*		
Soaping inhaler, dry with towel	15	(36)
Cleaning inhaler in dishwasher	2	(5)
No cleaning at all	4	(10)

*Total*	41	(100)

## Discussion

Despite decades of experience with inhaler therapy, a variety of mistakes concerning therapy adherence, the inhalation technique, and mistakes in the handling of spacer and device were made by Dutch children. The important role of the parents in all of these aspects is highlighted in this discussion.

### Therapy inconsistency

To determine therapy inconsistency we compared the reported use with the pharmacy prescription label, in our opinion the most appropriate source for comparison. The use of more than one inhaler was most frequently the reason for inappropriate use. Parents decide when and how the inhaler is to be used for most of the children, which confirms their important role in compliance with inhaler therapy. Noteworthy is that four children only used their ICS in case of symptoms, although ICS are a long-term maintenance therapy and should be administered daily.

### Inhalation technique

We found that many subjects did not shake their inhaler (c.f. Kamps et al)[6]. However, despite their mistake, most subjects knew that the inhaler had to be shaken before use. On the other hand, nineteen subjects did not know that it is recommended to activate the MDI once, inhale the first dose of drug, and then activate the MDI again for a second dose [16].

Most of the parents received the instruction either at the pharmacy or the general practice, emphasizing that both organisations play an important role in educating parents about the inhalation technique.

### Inhaler management

Most parents reported an incorrect method of assessing an inhaler for remaining doses. Most of them spray in the air to see whether the inhaler still contains gas. Fifteen subjects reported that they clean and dry their spacer with a towel. This is incorrect because the generated static load will prevent the drug from leaving the spacer for the first few inhalations [16]. Four subjects reported that they never cleaned their spacer.

### Is every mistake a failure?

Dutch children and their parents make a variety of mistakes when using an inhaler. However, despite the mistakes made, none of the children included in this study had severe asthma symptoms. It is therefore important to further investigate the effect of both the inhalation technique and the therapy consistency on asthma control. Especially ICS have become very potent in the last decade and even with a less correct inhalation technique relief of symptoms might be achieved.

### Strengths and limitations

This present study has several potential limitations. Although our strategy of using self-reports may not be ideal, no feasible alternative is available [8,10,12]. During the technique demonstration, some of the younger children became shy, which probably influenced their inhalation technique. The limited number of general practices and pharmacies may have affected the results. The proportion of responders, although low, is not uncommon in this setting [18]. As responders were probably more compliant, given their interest in the study, the overall compliance and inhaler technique might be even worse than our results suggest.

Strength of the present study is that the children were visited at home allowing them to demonstrate their inhalation technique in a familiar environment. We also explored knowledge on the inhalation technique and use, and combined these findings with the prescription.

## Conclusion

Children and their parents still make a variety of mistakes when using an inhaler. Concerning the inhalation technique, some easy to avoid mistakes were made, e.g. shaking and activating twice an inhaler before use. Therapy adherence was not optimal, especially when more than one inhaler was prescribed. In addition, mistakes were made related to cleaning the spacer and assessing the inhaler for remaining doses.

### Practice implications

Inadequate knowledge of when and how to use prescribed medication is one of the major barriers in achieving asthma control [19]. It is important that the GP give appropriate and written instruction to the parents, who play a prominent and important role in compliance with therapy of their children.

First of all, the inhalation technique should be clearly explained and well demonstrated. Preferably, this should be checked again during follow-up appointments to correct mistakes. Therapy regimen should be discussed, particularly when more than one inhaler is prescribed. An explanation of the difference between maintenance therapy and rescue medication is essential. We recommend further studies into the effect of education and monitoring on the appropriateness of inhalation technique in children (c.f. Haynes et al.[20]).

## Competing interests

The authors declare that they have no competing interests.

## Authors' contributions

JHJMU participated in the design of the study and helped to draft the text. YvU did the fieldwork and drafted the text. JCvdW participated in the design of the study and helped to draft the text. PJEB helped to draft the text. All authors read and approved the final manuscript.

## Funding body

The design, data collection, analysis, the writing of data and writing of the manuscript reported in this paper was made possible through internal funding of the department of General Practice, Erasmus Mc-University Medical Center Rotterdam.

## Supplementary Material

Additional file 1 **Inhalation technique questionnaire and general questionnaire items**. With these questionnaire items, the appropriateness of the inhalation technique was assessed. Click here for file
