# Supplementary material for: Adequate use of asthma inhalation medication in children: more involvement of the parents seems useful
Source: BMC Res Notes. 2009 Jul 13;2:129. doi: 10.1186/1756-0500-2-129 (PMC2718905; doi:10.1186/1756-0500-2-129)
Supplement: Additional file 1 — Inhalation technique questionnaire and general questionnaire items. With these questionnaire items, the appropriateness of the inhalation technique was assessed. [file 1756-0500-2-129-S1.doc]

**Adequate use of asthma inhalation medication in children: more involvement of the parents seems useful.** Johannes H.J.M. Uijen, Yannick J.W. van Uijthoven, Johannes C. van der Wouden, Patrick J.E. Bindels

**Additional file: Inhalation technique questionnaire and general questionnaire items**

*Inhalation technique questionnaire items:*

- Should the inhaler be shaken before use?
  - Yes
  - No
- Where on the face should the inhaler / the mask be placed?
  - Mouth
  - Nose
  - Both
- Should the mask firmly fit the face?
  - Yes
  - No
- How many doses should be brought into the spacer?
- At what speed does your child need to breath through the inhaler / the spacer?
  - Quick ( < 2 seconds)
  - Normal (2 – 5 seconds)
  - Slow ( > 5 seconds)
- How many times should your child breathe in and out through the inhaler / the spacer?
- When in need of multiple doses, how are these doses applied?
  - Initially bring all the doses in the spacer, then start the inhalation process.
  - Bring one dose in the spacer, then start inhalation process. Bring another dose in the spacer, and then repeat the inhalation process.
- Does your child need to rinse the mouth after inhaling?
  - Yes
  - No

*General questionnaire items:*

- Who provided instructions regarding the inhalation technique?
  - General practitioner
  - Pharmacy
  - Hospital
  - Somebody else
  - No instructions
- Was the inhalation technique checked during a follow-up appointment?
  - Yes
  - No
- Did you read the information leaflet provided with the inhaler?
  - Yes
  - No
- How do you assess the inhaler(s) for remaining doses?

………….

- How do you clean the inhaler(s) or spacer?
  ………….
- When does your child use the inhaler(s)? For example daily or as needed?
  ………….
- Who decides when the inhaler has to be used: you or your child?
  ………….
